# Female attractiveness affects paternal investment: experimental evidence for male differential allocation in blue tits

**DOI:** 10.1186/1742-9994-9-14

**Published:** 2012-06-25

**Authors:** Katharina Mahr, Matteo Griggio, Michela Granatiero, Herbert Hoi

**Affiliations:** 1 Konrad Lorenz Institute of Ethology (KLIVV), Department of Integrative Biology and Evolution, University of Veterinary Medicine, Vienna,Savoyenstraße 1a, A-1160, Vienna, Austria

**Keywords:** Female ornamentation, Male allocation, Parental care, Ultraviolet colouration

## Abstract

**Introduction:**

The differential allocation hypothesis (DAH) predicts that individuals should adjust their parental investment to their current mate’s quality. Although in principle the DAH holds for both sexes, male adjustment of parental investment has only been tested in a few experimental studies, revealing contradictory results. We conducted a field experiment to test whether male blue tits (*Cyanistes caeruleus*) allocate their parental effort in relation to female ornamentation (ultraviolet colouration of the crown), as predicted by the DAH.

**Results:**

We reduced the UV reflectance in a sample of females and compared parental care by their mates with that of males paired to sham-manipulated control females. As predicted by the DAH our results demonstrate that males paired with UV-reduced females invested less in feeding effort but did not defend the chicks less than males paired with control females.

**Conclusions:**

To our knowledge, this is one of the first studies providing support for male differential allocation in response to female ornamentation.

## Introduction

Females frequently choose males on the basis of traits [1] that may signal individual quality [2]. As a consequence they gain direct benefits, e.g. through high-quality territories and paternal investment, or indirect benefits, because attractive mates may provide genes for passing viability and attractiveness to the offspring [2]. On the other hand little is known about why females of several species also possess elaborate traits [3,4]. For a long time the presence of female ornaments was interpreted as being the consequence of genetic correlation with male ornamentation [3,5]. However, recent studies have suggested that female ornaments play a role in female - female competition (intrasexual selection) [6-10] or are sexually selected by males (intersexual selection) [11-17]. In species with biparental care males may gain benefits from choosing “high quality females” and adjust their parental investment to female quality. This may happen if there is much variance in female quality, if the latter affects offspring survival and if the males parental provide some parental investment and/or remating opportunities are low [2]. The idea of adjusting parental effort in response to the aesthetic traits of partners, when they represent honest signals of quality, is known as the Differential Allocation Hypothesis (DAH). Differential allocation is expected whenever individuals face a trade-off between current and future reproduction and the reproductive value of the offspring is connected to the attractiveness of the mate [18,19]. Since its original formulation, the DAH has been tested and supported in a number of taxa possessing different attractiveness traits and levels of parental care [20] but such studies have almost exclusively related female breeding investment to male attractiveness [21]. Given that female traits can indicate quality [9,11,14,22,23], males may differentially allocate parental investment in response to female attractiveness [24,25]. Some studies have explored the importance of female ornaments in male mate choice and as possible signals of female quality but very few have considered how they influence male parental investment [9,21,24,25].

The differential allocation of parental investment by males according to female attractiveness was tested in Burley’s [19] original experiments with captive zebra finches, *Taeniopygia guttata*. Male zebra finches paired with females manipulated to be more attractive (the manipulated trait was a pair of black leg rings) showed higher parental expenditure, in terms of feeding effort, than males paired with unattractive females [19]. Even if this was the first explicit test of the DAH, a previous study by the same author found an effect of male band color, but not female band color, on offspring weight, which one might expect is related to parental investment [18]. Only a few subsequent studies have investigated the DAH in relation to female ornamentation, producing contradictory results [19-21,24]. To our knowledge no study has replicated Burley’s results on paternal feeding effort in response to female ornamentation. Indeed, studies on rock sparrows (*Petronia petronia*) only partially supported the DAH as brood defence behaviour but not feeding investment was affected by female ornamentation [25]. Recently, a study on Gouldian finches (*Erythrura gouldiae*) found evidence for female, but not male, differential allocation in relation to mate quality [26].

In order to investigate whether males allocate their parental effort in response to female attractiveness we conducted a field experiment on the ultraviolet/blue crown coloration of blue tits (*Cyanistes caeruleus*). Previous studies have indicated that females adjust sex ratio, egg quality, feeding effort and nest defence behaviour in relation to the crown ultraviolet reflectance of mates [27-31]. Moreover, blue tits mate assortatively with respect to the UV reflectance of the crown [32,33] and previous studies found that female UV coloration was positively correlated with female survival, reproductive capacity and social status [10,14,34-37].

We captured breeding females during the chick-feeding stage and randomly assigned them to a control group, in which the female crown was smeared with duck preen gland oil alone, and UV-reduced group, in which the female crown was smeared with duck preen gland oil and UV-blocking chemicals. We recorded parental effort by conducting behavioural observations and measurements of nestling body mass. We hypothesized that in line with the DAH [18,19] males should allocate less parental care when mated to females with less UV reflectance as these may be expected to produce lower-quality offspring.

## Results

### Treatment

Before manipulation there was no significant difference in female UV chroma (Means ± SE: control = 0.50 ± 0.03, UV-reduced = 0.46 ± 0.03; *t*-test: *t* = 0.92, *P* = 0.36), body condition (control = 25.64 ± 0.85, UV-reduced = 24.10 ± 0.72; *t*-test: *t* = 1.36, *P* = 0.19) or wing-chord length (control = 6.64 ± 0.05; UV-reduced = 6.59 ± 0.07; *t*-test: *t* = 0.42, *P* = 0.68), laying date (control = 100.63 ± 1.41; UV-reduced = 101.89 ± 0.64; *t*-test: *t* = −1.44, *P* = 0.16) and brood size (control = 6.55 ± 0.51; UV-reduced = 7.58 ± 0.46; *t*-test: *t* = −0.92, *P* = 0.36) between the control (n = 11) and UV-reduced group (n = 19). The UV chroma of the crown plumage of male and female blue tits from 19 breeding pairs (10 control and 9 UV-reduced pairs), for which both male and female UV chroma was known, was positively correlated (*r* = 0.73, *n* = 19, *P* < 0.01), confirming the occurrence of assortative mating with respect to the UV-reflectance of the blue crown.

The reflectance spectra (UV chroma) of the crown was strongly affected by the treatment in the UV-reduced group (before = 0.46 ± 0.03, after = 0.40 ± 0.03, paired t- test: *t* = 9.60, *P* < 0.01), but not in the control group (before = 0.50 ± 0.03, after = 0.51 ± 0.03; paired *t*-test: *t* = −1.59, *P* = 0.14). This corresponded to a slight enhancement of UV chroma of 1.76% in the control group, or an average reduction of 13.36% in the treatment group.

### Treatment effects on parental effort

**Figure 1:**
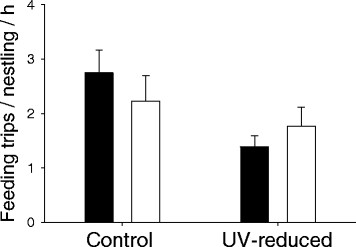
**Effect of female UV manipulation on the number of feeding trips per nestling per hour, performed by male (black bars) and female (white bars), whiskers show SE.** For clarity original values (not standardised) are shown.

**Figure 2:**
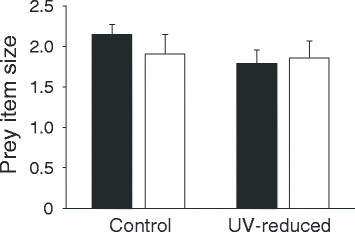
**Effect of female UV manipulation on the prey item size (calculated using the bill length, for more details see Methods), provided by male (black bars) and female (white bars), whiskers show SE.** For clarity original values (not standardised) are shown.

The final model revealed a significant treatment effect on the absolute number of feeding trips per nestling per hour (Table 1) but not on the relative number of feeding trips per nestling (effect size: 0.63; 95% CI: 0.24–1.02; *F*_*1,28*_ = 2.76, *B ± SE* = 0.29 ± 0.18, *P* = 0.11). Females in the UV-reduced and control group showed no difference in the number of feeding trips per hour (Standardised Means ± SE: control = −0.05 ± 0.28, n = 11; UV-reduced = 0.26 ± 0.24, *n* = 19; *t*-test: *t* = −0.19, *P* = 0.85) (Figure 1). Neither the absolute (effect size: 0.08; CI: -0.30–0.46; *F*_*1,28*_ = 0.04, *B ± SE* = −0.06 ± 0.19, *P* = 0.85), nor the relative number of female feeding trips per nestling per hour were affected by treatment (effect size: 0.31; 95% CI: -0.07–0.69; *F*_*1,26*_ = 0.65, *B ± SE* = −0.15 ± 0.18, *P* = 0.43). Average prey item size was incorporated in the model, demonstrating no significant effects of treatment (Figure 2), but there was a strong, but not significant, interaction between treatment and median laying date (Table 2). Further analyses of this effect show a trend towards a negative correlation between laying-date and average prey item size in males paired to UV-reduced females (*r* = −0.42, *n* = 19, *P* = 0.07), which does not occur in the control group (*r* = 0.46, *n* = 10, *P* = 0.18). Furthermore treatment was not retained in the final model for females, indicating the lack of a significant effect on female average prey item size, but the model revealed a significant effect of laying date on average prey item size (*F*_*1,22*_ = 5,8, *B* ± SE = −0.45 ± 0.19, *P* = 0.02). This might be due to a negative correlation between laying date and average prey item size in the female UV-reduced group (*r* = −0.47, *n* = 19, *P* = 0.04), this effect was not found in the control group (*r* = −0.38, *n* = 10, *P* = 0.27). We excluded one male and one female (both from the control group) from the analyses, because prey item size was not clearly visible during observation.

**Table 1:** Determinants of male feeding trips per nestling (treatment n = 19, control n = 11) (variables retained in the final model are in bold)

	***df***	***F***	***B* ± SE**	***p***
**Treatment**	**1, 28**	**5.43**	**0.39 ± 0.17**	**0.03**
Brood Size		0.01	−0.03 ± 0.19	0.89
Female UV Chroma		0.34	−0.10 ± 0.18	0.56
Egg-Laying Date		0.11	−0.06 ± 0.19	0.74
Treatment*Egg Laying Date		4.25	−0.38 ± 0.18	0.05
Treatment*Brood Size		0.96	−0.19 ± 0.19	0.33
Treatment*Female UV Chroma		0.11	−0.06 ± 0.18	0.74

**Table 2:** Determinants of male average prey item size (treatment n = 18, control n = 11)

	***df***	***F***	***B* ± SE**	***p***
Treatment		1.22	0.22 ± 0.19	0.28
Brood Size		0.02	−0.03 ± 0.24	0.61
Female UV Chroma		0.12	−0.07 ± 0.21	0.90
Egg-Laying Date		0.26	−0.10 ± 0.20	0.61
Treatment*Egg Laying Date	1,22	3.43	0.37 ± 0.19	0.08
Treatment*Brood Size		1.15	−0.25 ± 0.24	0.29
Treatment*Female UV Chroma		1.06	0.22 ± 0.21	0.32

We found no statistically significant differences between the average body mass of nestlings from nests of control or UV-reduced females (Standardised Means ± SE: control = 0.05 ± 1.06, *n* = 10; UV-reduced = −0.03 ± 0.93, *n* = 18; *t*-test: *t* = 0.23, *P* = 0.53). Nestlings from one brood of the control group and one brood of UV-reduced group were not measured due to adverse weather conditions. Brood size and the start of egg laying were retained in the final model (Table 3) and therefore seem to explain some of the variation in average nestling body mass between nests. Female UV chroma was also retained in the model, suggesting a weak negative effect of female UV reflectance of the female’s crown on nestling body mass.

**Table 3:** **Determinants of nestlings body mass (*n*** **= 28) (variables retained in the final model are in bold)**

	***df***	***F***	***B* ± SE**	***p***
Treatment		0.03	0.02 ± 0.15	0.87
**Brood Size**	**1, 20**	**9.10**	**−0.59 ± 0.18**	**<0.01**
**Female UV Chroma**	**1, 20**	**1.80**	**−0.41 ± 0.17**	**0.03**
**Egg-Laying Date**	**1, 20**	**11.95**	**0.64 ± 0.18**	**<0.01**
Treatment*Egg Laying Date		0.28	0.09 ± 0.18	0.61
Treatment*Brood Size		0.46	−0.12 ± 0.18	0.50
Treatment*Female UV Chroma		0.91	−0.17 ± 0.18	0.35

### Treatment effect on nest defence behaviour

Using Mann Whitney U-Tests we found no statistically significant differences in the three variables (latency, time of rattling, number of attacks) describing nest defence behaviour between males paired with control and UV-reduced females (Table 4). Furthermore, no behavioural differences related to nest defence behaviour were found between females from the control or UV-reduced group (Table 4).

**Table 4:** Differences in the three variables describing nest defence behaviour tested for males and females from either the control (C) or UV-reduced (UV-) group (z-transformed data, see Methods)

	**UV- (n =13)**	**C (n = 8)**	**Mann–Whitney *U*-test**
Male latency	−0.46	−0.45	*U* = 47.5, *p* = 0.74
Male rattling	−0.62	0.41	*U* = 34.5, *p* = 0.21
Male attacks	−0.19	−0.56	*U* = 37.5, *p* = 0.29
Female latency	−0.31	−0.32	*U* = 46.0*, p* = 0.66
Female rattling	−0.12	−0.15	*U* = 46.0*, p* = 0.66
Female attacks	−0.32	−0.29	*U* = 50.0*, p* = 0.88

## Discussion

The UV reflectance of the crown plumage of female blue tits significantly affected male investment in feeding nestlings. Males made less frequent feeding trips when paired with UV-reduced females, as predicted by Burley [18]. The original DAH predicts that individuals face a trade-off between current and future reproduction and therefore adjust their parental effort in accordance with their mate’s aesthetic traits, whenever these traits are linked to the mate’s quality [18]. This is based to the assumption that heritable quality (“good genes”) will increase offspring value and eventually result in more grand offspring and, consequently, higher fitness. Consequently males should invest more in offspring provisioning when paired with ornamented females if male provide parental care and female quality strongly affects offspring survival.

UV coloration is thought to be an indicator of individual quality and to be a sexually selected trait (but see [38]). The UV reflectance of structural feathers is determined by a combination of pigments and nanometre-scale structures [39,40]. Recent studies suggested that fast moulting and nutritional stress can affect the colours of structural feathers [37]. However, a recent experimental study found no evidence for a relationship between nutritional and body condition and UV coloration in blue tits [41]. Recent studies have revealed that the UV reflectance of structural feathers is sensitive to wear and might easily be affected by the accumulation of dirt and parasites [42-45]. Individuals need to invest time in feather maintenance to keep their feathers in good condition. Plumage maintenance is a costly and time-consuming process, which forces individuals into a trade-off between plumage maintenance and time they need for other activities (e.g. foraging) [46,47]. Hence, it could be that only individuals in good condition can afford these costs and therefore high UV reflectance [48].

Our results and recent studies suggest an association between female blue tit UV coloration and condition as well as female breeding performance [14,34-37]. Therefore, we consider UV reflectance to be a potential indicator trait that might be used by males to assess female quality. Even though a decrease in UV reflectance during the breeding season is a normal process [36] the sudden reduction caused by our treatment might indicate a drop in the condition and parental quality of the current mate. Given that food provisioning for the offspring is costly for the feeding parent [49], our data suggest that males differentially allocate feeding investment to enhance their chances of future reproduction. Females in bad condition might not be able to provide sufficient parental care, which in turn affects nestling body mass and growth. A study on ring-necked pheasants (*Phasianus colchicus*) revealed that selected adult sexual ornamentation can reflect nutritional condition during early phases of growth [50]. Furthermore, recent studies on blue tits indicate that the UV coloration of nestlings is affected by condition [51,52]. Juvenile males in better condition appear to develop more colourful tail feathers [52] that are not moulted during the post-juvenile moult [53] and therefore might enhance attractiveness in the first breeding year.

According to the basic idea that individuals choose mates on the basis of ornaments that reflect quality, males paired with females in poor condition might face the problem of producing offspring with low reproductive success. In other words, because of reduced female quality the reproductive value of the offspring will be reduced, also from the male’s perspective. The difference in male feeding investment in response to the female treatment is therefore consistent with the prediction that DA is strongly related to reproductive life-history. Our results on paternal care investment complement other studies, which demonstrated the female’s differential allocation of parental care in blue tits in response to the manipulation of male UV coloration of the crown [28,29]. Both studies indicate that male UV coloration is under selection pressure, created by the female allocation of parental care. Based on our results and other studies [32,33], which likewise showed assortative mating in response to UV reflectance, we suggest that male differential allocation may affect selection on female UV coloration.

Whereas the original concept of the DAH also suggests that less attractive partners should increase their parental investment [18,19], we found no effects of the treatment on female parental care. Johnsen et al. [29] demonstrated that males invested more into parental care when their UV reflectance was reduced. One possible explanation for the lack of compensation by females could be that they were already making the maximum possible investment [54]. The weather during spring 2009 and especially 2010 was cold and rainy. Food resources for the birds were probably limited and female food provisioning was restricted by the availability of resources. Although males provided significantly less food to nestlings from UV-reduced females than to females from the control group, and females did not compensate for this lack of feeding effort, we found no effect of treatment on body mass in either group. One reason could be that the experiment was conducted only a few days before fledging, a time during which passerine nestlings often lose weight [55]. Furthermore, daily data on nestling development were not collected after female treatment, although this could have been informative.

The experimental approach of Burley [19] revealed a correlation between female attractiveness and male feeding investment in zebra finches, whereas no relationship was found between the attractiveness of the female and the nest defence behaviour of their mates. A recent experiment on rock sparrows showed a reduction in male nest defence intensity in response to a reduction in female attractiveness, but not in feeding investment [24,25]. On the contrary, here we found no differences in the nest defence behaviour of male blue tits facing either control or UV-reduced females. Our results may reflect the lack of treatment effect during the nest defence experiment. The reduction in UV reflectance was only small (13.36%), with the aim of avoiding a confounding “strange-mate” effect. The treatment is known to diminish UV reflectance for at least 5 days in wild birds [56]. Whereas the feeding investment observation was conducted the day after manipulation of the female crown plumage, nest defence behaviour was observed 3–4 days after treatment. During this period the UV-reducing chemical could have been removed by plumage maintenance. Further research is required to test whether male differential allocation only takes place during certain stages of parental investment and to test in which stages of the breeding cycle male allocation occurs.

In conclusion, our results indicate that male blue tits make parental care decisions in accordance with their mate’s quality. To our knowledge this is one of very few experimental studies demonstrating male differential allocation in relation to female attractiveness.

## Methods

### General methods

The study area is located in Pressbaum, near Vienna (48° 18´ N, 16° 8´ E; about 320 m a.s.l.). Experiments were carried out in two consecutive breeding-seasons (2009, 2010). Approximately 250 nestboxes were installed in 2008, and these were monitored every 3 days from the beginning of March until mid June.

On day 11 (±1) after the nestlings had hatched, parent blue tits were captured on the nest by closing the entrance hole of the nest-box. Sex was determined according to the breeding patch, which was still clearly visible, and was confirmed by carrying out sexing PCRs (see below) after the breeding season. Crown colouration was measured (see below) after sexing the birds and the birds were then banded with aluminium rings and a unique combination of darvic colour rings. Standard measurements of the flattened wing chord length were taken to the nearest 0.5 mm. Weight was recorded to the nearest 0.1 g [57]. After the measurements a blood sample (25 μl) was taken from the brachial vein, from both adults and nestlings. The procedure was completed in less than 25 min to ensure sufficient feeding of the nestlings. Nestlings’ body mass was recorded to the nearest 0.1 g on day 10 and 12 (±1 day) post-hatching. Nestlings were ringed and measured on approximately day 15 post-hatch.

### Molecular sexing

Sexing-PCR amplifications were carried out in a total volume of 1.5 μl. Conditions were as follows: 1X PCR Buffer, 2.5 mM M_g_Cl_2_, 200 μM dNTPs, 0.1 uM of each primer (P2, P8), 2.5 U/ul FirePol, distilled water and 2 μl DNA [58]. The PCR was performed in a programmable T1 Thermocycler (Biometra, Göttingen, Germany). Separation was achieved via gel-electrophoresis for 45–50 min at 9–10 V⋅cm^-1^, in a 2.5% agarose gel [58].

### Treatment and spectrometry

The crown coloration of captured males and females was measured using a USB-2000 spectrometer and a DHS-2000-FHS deuterium halogen lamp, connected through a bifurcated fibre-optic probe (Ocean Optics, Eerbek, The Netherlands). To exclude disturbance by outer light sources and to ensure a standardized distance and angle (90°), a black rubber cylinder was fitted to the top of the probe. Before each measurement the spectrophotometer was recalibrated using a standard white (Avantes, Eerbek, The Netherlands); for calibration of black the probe was removed from the light source and the cap of the plug closed. Standard descriptors of reflectance spectra were used for quantification of colors. Measurements were taken from five spots on the crown plumage. Calculations were carried out for reflectance in the 300–700 nm range. To quantify the UV reflectance of the blue crown plumage we chose the variable UV chroma, which is defined as the proportion of UV reflectance out of total reflectance (R_300_ – R_400_/R_300_ – R_700_) [36,37,44,59,60]. Pairs were randomly assigned to UV-reduced or control groups (control: n = 11, UV-reduced: n = 19). No statistical differences were detected between the two groups for laying date and brood size (see Results). The UV reflectance of the crown plumage was reduced in the UV-reduced group using a 40/60% (w/w) mixture of UV-blocking chemicals (50/50 w/w blend of Parsol 1989 and MCX, Roche Switzerland) and duck preen gland fat; the control group was treated with duck preen gland fat alone [46]. After the feathers had dried, the reflectance of the crown plumage was measured as described above. The chemicals employed are commonly used to reduce the UV reflectance of plumage and are known to have no negative effect on bird health and behaviour [48].

### Feeding investment observations

On day 13 (± 1) post-hatching, after manipulating the color of the female, nests were observed with a spotting telescope for 1 h, either between 0600 and 1100 or 1500 and 1700 h when the feeding rate was the highest (own observation). The observer was blind to the group (treatment/control) and remained about 20 m away from the nest box, to avoid influencing feeding behaviour. The number of feeding trips and average prey item size were recorded for each parent. Prey item size (or food size when more than one prey item was carried) was estimated by comparing bill length with prey length. Based on similar studies prey item size was then categorized in three classes: (1) as long as one bill length and smaller, (2) longer than one bill length but smaller than two bill lengths and (3) two bill lengths or larger [25]. Average prey item size was defined as the sum of observed size categories divided by the feeding trips the individual performed during one hour of observation. Feeding trips were quantified as the number of visits to the nest per nestling during one hour of observation. For these variables we also calculated the relative values (feeding trips or food load of one parent/feeding trips or food load of both parents). Relative values were used because maternal and paternal investment might have been correlated.

### Nest defence behaviour

To investigate, whether reducing the female UV reflectance influenced male nest defence behaviour, a trial was performed on the last day of the experiment (13–14 days post-hatching). To simulate predation by a common terrestrial predator, the aesculapian snake (*Zamenis longissimus)*, a rubber dummy of a snake was put with the head in the hole of the nest-box and the rear-body was placed on the roof of the nest-box. The dummy was positioned while both parents were away from the nest-box. The aesculapian snake is known to be an important predator of nestlings in our study area [29]. Following the arrival of the first adult individual, the birds were observed for 15 min. During this time the number of attacks and the time individuals spent around the dummy predator were recorded. Latency time was defined as the time span between placing the dummy and the arrival of the first member of the pair. All values concerning time were recorded in seconds [25,61].

### Statistical methods

All statistical analyses were performed using Statistica 7.1 (Statsoft Inc., Tulsa). The data were tested for normal distribution, and statistical analyses were conducted as appropriate. Independent t-tests between the control and UV-reduced group were used to test for possible differences in female wing chord length, body condition (body condition was calculated by dividing body mass by (tarsus length) ^3^ owing to small values the body condition indices were multiplied by 10^4^) [62] and UV chroma before manipulation, laying date and brood size. To test for treatment effects on parental feeding investment and nestling body mass, we used General Linear Models (GLM). We included original female UV chroma as the covariate in the initial models, because UV chroma has been found to correlate with measures of female quality [14,31,35-37]. The initial models included the start of egg laying and brood size on day 13 (±1), respectively, to control for the effects of these variables on the feeding performance of the birds [29]. The initial models also included the interactions between each response variable and treatment. Starting with the interactions, non-significant terms were eliminated from the model step-by-step. Main effect terms were retained in the model while testing for the interaction effect. Each eliminated term was reentered into the final model to confirm the lack of significance [63]. We found significant differences in female feeding effort between the two study years. To avoid the loss of statistical power, by incorporation of an additional variable into the GLM, we decided to correct for annual effects by using a z-standardization ((value-mean of the year)/standard deviation of the year) [63]. Models testing effects on feeding investment were conducted with absolute and relative values (feeding trips of one parent/feeding trips of both parents). *P*-values below 0.05 were considered to be significant and all values for parametric tests are given as Mean±Standard Error (SE); for non-parametric tests values are given as the Median.

## Ethics statement

The experiments reported in this paper comply with the current laws on animal experimentation in Austria and the European Union. The long-term nature of the study allowed us to confirm that handled birds and their offspring did not suffer any detectable reduction in welfare and survival.

## Competing interests

The authors declare that they have no competing interests.

## Authors’ contributions

All authors collected the data, analyzed the data and wrote the paper. All authors read and approved the final version of the manuscript.
